# Omitting anthracyclines for the adjuvant treatment of patients with triple-negative breast cancer: A non-inferiority meta-analysis

**DOI:** 10.1016/j.breast.2025.104524

**Published:** 2025-06-30

**Authors:** Fabio Girardi, Caterina Barbieri, Gaia Griguolo, Daniela Iannaccone, Christian Zurlo, Maria Vittoria Dieci, Valentina Guarneri

**Affiliations:** a Division of Medical Oncology 2, Veneto Institute of Oncology IOV-IRCCS, Via Gattamelata 64, Padua, 35128, Italy; b Department of Surgery, Oncology and Gastroenterology, University of Padua, Via Giustiniani 2, Padua, 35121, Italy

**Keywords:** Triple-negative breast cancer, Adjuvant, Chemotherapy, Anthracycline-free, Metanalysis

## Abstract

**Introduction:**

For patients diagnosed with triple-negative breast cancer (TNBC), the sequential use of anthracyclines and taxanes is the standard adjuvant treatment, when this is indicated. However, anthracycline-related toxicities represent a concern. We conducted a meta-analysis to assess whether anthracycline-free regimens are non-inferior to standard, sequential regimens.

**Patients and methods:**

We used a complex search strategy to query multiple databases. The population included patients who underwent primary surgery for TNBC, eligible for adjuvant chemotherapy and randomised in a phase 2 or 3 clinical trial. We fitted non-inferiority (NI) margins using published treatment effects. We calculated risk ratios (RR) for recurrence or death.

**Results:**

Eight studies out of 3410 potentially eligible records were included in the meta-analysis, for an overall population of 4292 patients. The RR for recurrence was 1.05 (95 % confidence interval (CI) 0.93–1.19), with an upper bound superimposing on the NI margin of 1.19. In a sensitivity analysis excluding the two studies using CMF, the recurrence RR for the comparison between taxane-only chemotherapy and anthracycline-based sequential chemotherapy was RR 0.97 (95 % CI 0.84–1.11). The RR for death was 1.17 (95 % CI 1.00–1.37), with an upper bound crossing the NI margin of 1.16.

**Conclusions:**

Anthracycline-free adjuvant chemotherapy may represent an option for patients with early TNBC who are not eligible for pre-operative treatment and for whom sparing anthracyclines should be considered (e.g., young patients with small tumours, patients at risk of adverse effects). Non-inferiority was more evident for taxane-only chemotherapy than for anthracycline-free regimens at large. However, our results call for caution considering the remarkable heterogeneity in the study patient populations. This meta-analysis should prompt further research into strategies for patient selection, including the use of prognostic biomarkers for risk stratification.

## Highlights

• The use of adjuvant anthracyclines may be associated with long-term toxicities.
• We assessed the impact of omitting adjuvant anthracyclines on outcomes of TNBC patients.
• Anthracycline-free chemotherapy proved to be non-inferior to sequential adjuvant chemotherapy, with the strongest, pooled effect being observed for taxane-only regimens.
• Non-inferiority was proved for the risk of recurrence, but not for the risk of death.
• Our findings may be relevant to a low-risk population undergoing upfront surgery, but caution is warranted given the heterogeneity of the studies.

## 1 Introduction

Triple-negative breast cancer (TNBC) is associated with an aggressive clinical behaviour. Five-year relative survival is 77 % for all stages combined, compared to 85 % or more for other breast cancer subtypes [[1], [2], [3]].

The Early Breast Cancer Trialists Collaborative Group (EBCTCG) patient-level meta-analysis on long-term outcome of 100,000 women diagnosed with early breast cancer, showed that for any anthracycline-based regimen, the risk of any recurrence was 27 % lower compared to no chemotherapy, and, for the same comparison, the benefit on the risk of overall mortality was up to 16 %. The benefit was consistent across subgroups defined by tumour stage, nodal stage, oestrogen receptor status (ER) or tumour grade [4].

For patients diagnosed with TNBC and receiving upfront surgery, adjuvant chemotherapy is the standard treatment protocol, based on the European Society for Medical Oncology (ESMO) guidelines, possibly excluding from this indication very early-stage, low-risk cases [5].

The sequential use of anthracyclines and taxanes became established as the standard adjuvant treatment protocol [5], based on trial and meta-analysis-level evidence [4,[6], [7], [8], [9]].

However, potential long-term cardiovascular or haematological toxicities following anthracycline treatment still represent a concern for clinicians [[10], [11], [12], [13]]. These toxicities are relatively rare, but they may impact life expectancy or quality of life. In a meta-analysis including 22,815 patients, after a median follow-up of nine years, six percent of patients had developed clinically overt cardiotoxicity, while for 18 % cardiotoxicity was only subclinical [14]. In a large retrospective study of 92,110 patients diagnosed with breast cancer, the incidence rate of acute myeloid leukaemia (AML) or myelodysplastic syndrome (MDS) was higher in patients receiving anthracyclines or sequential chemotherapy (around 1 and 2 new cases per 1000 person-years for AML and MDS, respectively) than in patients treated with docetaxel plus cyclophosphamide (TC) or cyclophosphamide plus methotrexate and fluorouracil (CMF) [6].

In connection with this evidence, several studies aimed to explore the benefit of using alternative, anthracycline-free treatment options for patients with human epidermal growth factor receptor (HER2) negative breast cancer. However, the findings were inconsistent. In the US Oncology (USO) Trial 9735, four cycles of TC were associated with an improved DFS, compared to four cycles of adriamycin plus cyclophosphamide (AC), after seven years of follow-up (81 versus 75 %; hazard ratio (HR) 0.74, 95 % confidence interval (CI) 0.56–0.98). The benefit was also seen for overall survival (OS), with a significant 31 % reduction in the risk of death [15]. Conversely, the Anthracyclines in Early Breast Cancer (ABC) trials compared six cycles of TC with one of several triple-drug regimens including doxorubicin, cyclophosphamide and a taxane. The long-term follow-up showed that triple-drug regimens were associated with a benefit in invasive DFS (iDFS) compared to taxane-only regimens (1.30, 95 % CI 1.02–1.67, p = 0.04) [16,17]. The non-inferiority West German Study Group (WGS) PlanB trial, comparing four cycles of epirubicin plus cyclophosphamide (EC) followed by four cycles of docetaxel with six cycles of TC, found no difference between the two arms [18]. Few trials were specifically designed for patients with TNBC. For instance, the Platinum and Taxane in Triple-Negative Breast Cancer (PATTERN) trial randomised 647 patients to receive either six cycles of weekly carboplatin plus paclitaxel, or fluorouracil plus epirubicin and cyclophosphamide (FEC) followed by docetaxel for six cycles in total. DFS was better for carboplatin-paclitaxel than for FEC-docetaxel (five-year DFS, 87 versus 80 %, HR = 0.65, 95 % CI 0.44–0.96), but no difference was found for OS [19].

Given the lack of robust and consistent evidence on treatment modulation for patients with early TNBC, we set out to conduct a non-inferiority meta-analysis to fill this gap in knowledge.

## 2 Methods

We queried the databases Embase, Medline, and PubMed to retrieve all potentially eligible publications. We also searched the American Society of Clinical Oncology (ASCO) and European Society for Medical Oncology (ESMO) web-proceedings.

We pre-specified the inclusion criteria, using the Patient, Intervention, Comparison, Outcome (PICO) model. The search strategy consisted of a combination of free-text words and exploded medical subject headings (Supplementary Tables 1, 2, and 3).

We were interested in randomised phase 2 or 3 clinical trials including patients diagnosed with early, HER2-negative breast cancer, comparing anthracycline-free with anthracycline-based treatment regimens. Eligible studies were not required to be specifically designed for patients with TNBC, but data for this population had to be provided.

We selected the records using a stepwise process: by title, abstract and, lastly, appraising the full text. Records were independently assessed by two reviewers. In case of disagreement, a consensus was reached through discussion. Each phase of the process was recorded and summarised following the Preferred Reporting Items for Systematic Reviews and Meta-analysis (PRISMA) guidelines.

For the meta-analysis, we chose a random-effect, non-inferiority (NI) design [20]. We regarded the anthracycline-free arm as the experimental arm to be tested, while the anthracycline-based arm was considered the active control. Based on the systematic review, we found wide variation between studies, which tested the hypothesis using either a superiority or a non-inferiority design. We overcame this issue by abstracting the study-level raw number of events to recalculate RRs and CIs around the point estimate. The outcomes of interest were the risk of recurrence and the risk of death.

We defined NI margins for each outcome of interest. We used as a reference the RRs and the corresponding CIs of the 2012 EBCTCG meta-analysis on adjuvant chemotherapy [4]. The NI margin was defined as the inverse of the treatment effect of sequential chemotherapy versus anthracycline-only chemotherapy.

We used the statistical package *metan* implemented in the software STATA17®.

## 3 Results

**Fig. 1:**
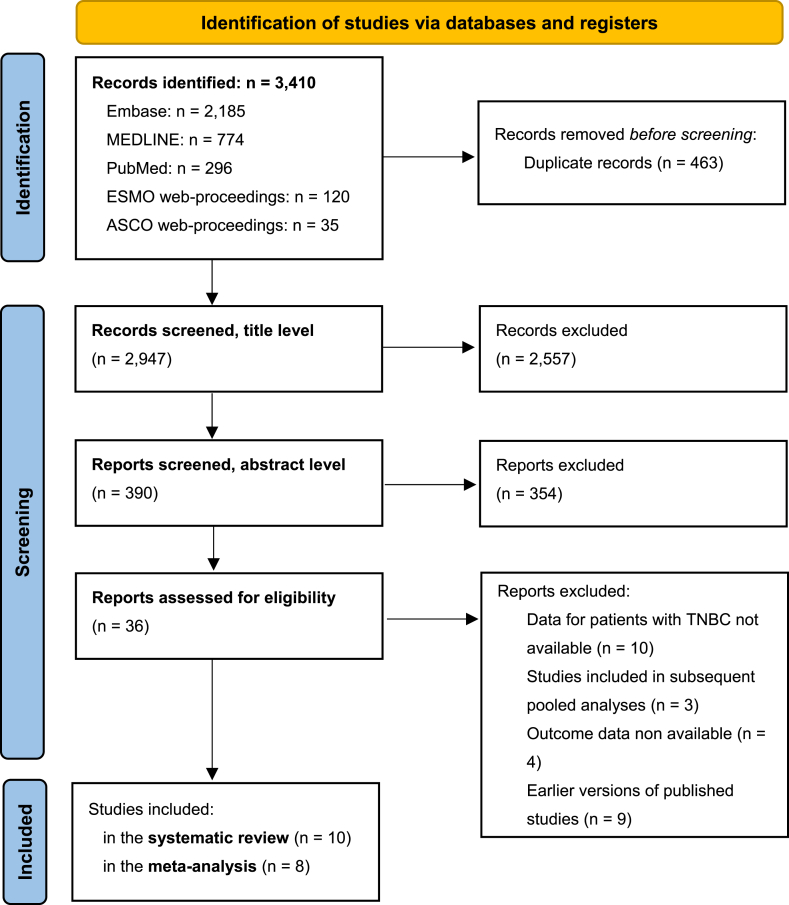
Preferred Reporting Items for Systematic Reviews and Meta-Analyses (PRISMA) flowchart. Modified, from: Page MJ, McKenzie JE, Bossuyt PM et al. The PRISMA 2020 statement: an updated guideline for reporting systematic reviews. BMJ 2021; 372: n71.

Using a database-specific, complex search strategy, we obtained 3410 potentially eligible records. After the selection process, ten studies met the criteria for inclusion in the systematic review (Fig. 1). These studies covered overall a population of 4416 patients diagnosed with TNBC.

Eligible studies were published between 2012 and 2024 and were conducted in Europe, North America, and Asia. Six studies used a superiority design, while four used a non-inferiority design. Only four studies were specifically designed for patients with triple-negative breast cancer. The median study follow-up was 67 months (Table 1).

**Table 1:** Characteristics of the eligible studies.

Study	Phase	Year	Design	Inclusion criteria	Patients (n)	Patients with TNBC (n)	Treatments arms	Median follow-up (months)
Earl HM [NEAT/BR9601]	3	2012	Superiority	Any patient requiring adjuvant chemotherapy	2391	385	E(4)>CMF(4) vs CMF(6,8)	89
Rocca A	3	2014	Superiority	pN1 or T ≥ 1 cm, N-neg	705	156	E(4)>CMF(4), CMF(4)>E(4) vs CMF(6)	69
Mavroudis D	3	2016	Non-inferiority	HER2-neg: N-pos	650	74	FEC(4)>D(4) vs TC(6)	46
Najafi S	2	2017	Superiority	TNBC	119	119	AC(4)>CBDCA+D(4) vs CBDCA+D(6)	40
Wang J	3	2019	Superiority	TNBC	132	132	ddEC(4)>P(4) vs dd(CBDCA+P)(4)	57
Yu KD [PATTERN]	3	2020	Superiority	TNBC: N-pos or T ≥ 1 cm, N0	647	647	FEC(3)>D(3) vs w(CBDCA+P)(6)	62
Yu KD [MASTER]	3	2021	Non-inferiority	HER2-neg: pT1-3, N-pos or pT2-3, pN0, HR-neg or pT2-3, pN0, G2-3 or pT2-3, pN0, LVI or pT2-3, pN0, ≤35 yo	1571	81	TC(6), FEC(3)>T(3) vs EC(4)>wP(12)	66
Zheng F	2	2021	Non-inferiority	TNBC	308	308	EC(4)>D(4), EC(4)>P(4) vs CBDCA+D(6), CBDCA+P(6)	97
De Gregorio A [PlanB, SUCCESS C]	3	2022	Superiority	HER2-neg: N-pos or T ≥ 2 cm, N-neg, HR-neg or T ≥ 2 cm, N-neg, uPA/PAI-1-high or T ≥ 2 cm, N-neg, ≤35 yo or T ≥ 2 cm, N-neg, G2-3	5924	1279	EC(4)>D(4), FEC(3)>D(3) vs TC(6)	62
Geyer CE [USOR 06–090, NSABP B-46-I/USOR 07132, and NSABP B-49]	3	2024	Non-inferiority	HER2-neg: pT1-3, N-pos or T ≥ 2 cm, pN0 or HR-neg, pN0 or pT1c, pN0, HR-pos, G3 or pT1c, pN0, HR-pos, high Oncotype RS	4181	1304	AC(4)>wP(12), ddAC(4)>wP(12), ddAC(4)>P(4), TAC(6) vs TC(6), TCB(6)	83

TNBC: triple-negative breast cancer; N: node; pN: pathological node staging; T: tumour; pT: pathological tumour staging; HER2: human epidermal growth factor 2; HR: hormone-receptor; pos: positive; neg: negative; RS: recurrence score; G: grade; LVI: lymphovascular invasion; uPA/PAI-1 = urokinase plasminogen activator/plasminogen activator inhibitor-1; yo: years old; CMF: cyclophosphamide, methotrexate, and 5-fluorouracil; E: epirubicin; FEC = 5-fluorouracil, epirubicin, and cyclophosphamide; EC: epirubicin and cyclophosphamide; ddEC: dose-dense epirubicin and cyclophosphamide; AC = doxorubicin and cyclophosphamide; ddAC: dose-dense doxorubicin and cyclophosphamide; D: docetaxel; P = paclitaxel; CBDCA = carboplatin; wP: weekly paclitaxel; w(CBDCA + P): weekly carboplatin and paclitaxel; dd(CBDCA + P): dose-dense carboplatin and paclitaxel; TC: docetaxel and cyclophosphamide; TAC: docetaxel, doxorubicin, and cyclophosphamide; TCB: docetaxel, cyclophosphamide, and bevacizumab. Numbers in brackets are the number of courses of a given chemotherapy regimen.

Anthracycline-free treatment arms varied between studies: CMF chemotherapy, carboplatin plus a taxane, or TC chemotherapy (Table 1).

In four studies, the anthracycline-free regimen did not prove non-inferior to the anthracycline-based regimen [16,[21], [22], [23]]. Two of the six studies using a superiority design found that the use of anthracyclines was associated with an improved outcome [24,25], while in four outcomes were similar or in favour of the anthracycline-free regimens [19,[26], [27], [28]]. (Table 1)

Outcome data by tumour or nodal stage were only available in few studies. In the PATTERN superiority study, disease-free survival (DFS) in patients receiving carboplatin and a taxane was better than in patients receiving sequential chemotherapy, but this treatment effect crossed one after stratification by tumour size, 2 cm or less and more than 2 cm [19]. Similarly, the difference between the two arms was not significant after stratification by number of involved lymph nodes, up to three and more than three [19]. In the study by Zheng et al., an adjuvant treatment regimen including carboplatin and a taxane proved to be non-inferior to sequential chemotherapy in terms of DFS, for both patients with node-negative and patients with node-positive disease [23]. Conversely, in the final pooled analysis of the ABC trials, after 6.9 years of follow-up, six cycles of TC chemotherapy were inferior to sequential chemotherapy for DFS, regardless of nodal stage [17].

In three studies, anthracycline-free regimens were associated with higher frequencies of febrile neutropenia, compared to anthracycline-based regimens [22,28,30]. Conversely, in the PATTERN study [19], febrile neutropenia was more common with FEC chemotherapy followed by docetaxel than with the carboplatin and paclitaxel combination. Peripheral neuropathy (any grade) was more common with anthracycline-free regimens than with anthracycline-based regimens in four studies [19,21,22,26], while a reverse pattern was observed in three studies [17,23,28]. Four cases of acute leukaemia were reported in the anthracycline-based treatment arms, three in the National Epirubicin Adjuvant Trial (NEAT)-BR9601 pooled analysis, and one in the Minus Anthracycline or Short-Term **versus** Epirubicin and Cyclophosphamide followed by Paclitaxel Regimen for Adjuvant Breast Cancer Therapy (MASTER) study [17,22,25]. An excess of any grade cardiac events with anthracyclines was only reported in the MASTER study [22]. (Supplementary Table 4)

We assessed study quality using the Cochrane's Rob 2 tool. Overall, the quality of the studies was good. Three out of the ten studies included in the systematic review raised some concerns regarding potential bias, but none were found to be highly biased.

Only eight studies were included in the meta-analysis, as the number of events was not available for two studies [21,26]. These eight studies covered a population of 4292 patients diagnosed with TNBC (96 % of the population covered by the systematic review).

A total of 2181 and 2111 patients received anthracycline-based or anthracycline-free regimens, respectively.

**Fig. 2:**
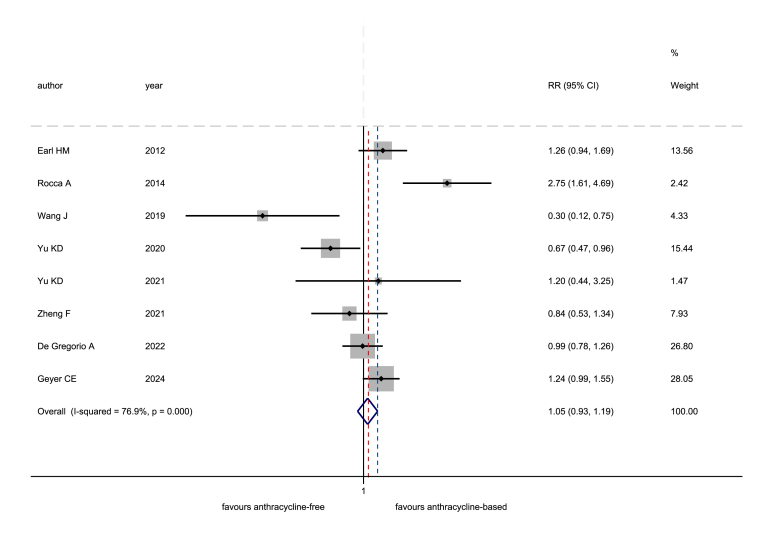
Non-inferiority meta-analysis for the risk of recurrence: comparison between anthracycline-free and anthracycline-based chemotherapy. RR: risk ratio; CI: confidence interval. The dotted, red line corresponds to the pooled treatment effect, while the dotted, blue line corresponds to the non-inferiority margin. (For interpretation of the references to colour in this figure legend, the reader is referred to the Web version of this article.)

**Fig. 3:**
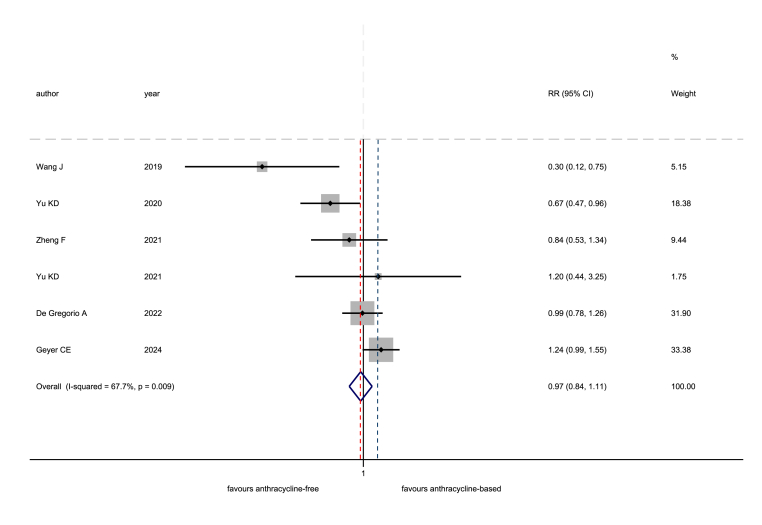
Non-inferiority meta-analysis for the risk of recurrence, after excluding trials using cyclophosphamide plus methotrexate and fluorouracil (CMF) chemotherapy as the experimental arm to be tested: comparison between taxane-based and anthracycline-based chemotherapy. RR: risk ratio; CI: confidence interval. The dotted, red line corresponds to the pooled treatment effect, while the dotted, blue line corresponds to the non-inferiority margin. (For interpretation of the references to colour in this figure legend, the reader is referred to the Web version of this article.)

In the anthracycline-based arms, 412 recurrence events occurred, while in the anthracycline-free arms the events were 416. The estimated pooled RR was 1.05 (95 % CI 0.93–1.19). The upper bound of the confidence interval overlaid the pre-defined NI margin of 1.19 (Fig. 2). The I^2^, a measure of between-study heterogeneity, was equal to 76.9 %. In a sensitivity analysis excluding the two studies using CMF chemotherapy as the anthracycline-free arm, the pooled RR was 0.97 (95 % CI 0.84–1.11), which lay within the NI margin (Fig. 3).

**Fig. 4:**
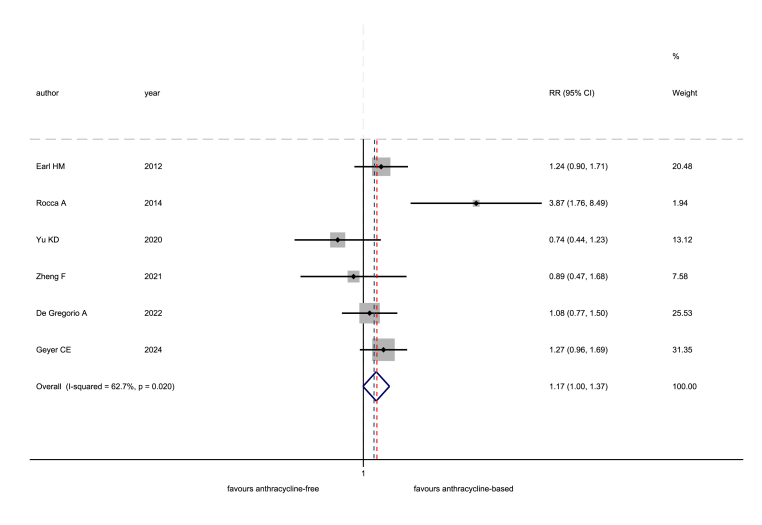
Non-inferiority meta-analysis for the risk of death: comparison between anthracycline-free and anthracycline-based chemotherapy. RR: risk ratio; CI: confidence interval. The dotted, red line corresponds to the pooled treatment effect, while the dotted, blue line corresponds to the non-inferiority margin. (For interpretation of the references to colour in this figure legend, the reader is referred to the Web version of this article.)

Only six studies allowed the analysis for the risk of death. Death events were 241 in the anthracycline-based arms, and 272 in the anthracycline-free arms. The estimated pooled RR was 1.17 (95 % CI 1.00–1.37), with the upper bound of the confidence interval crossing the NI margin of 1.16. The I^2^ was equal to 62.7 % (Fig. 4). After excluding the two CMF studies, the results were similar, but the analysis was based on only four studies (data not shown).

## 4 Discussion

To our knowledge, this is the first meta-analysis to date on the omission of anthracyclines in the adjuvant treatment of patients diagnosed with early TNBC. We found that anthracycline-free regimens were non-inferior to anthracycline-based, sequential regimens for the risk of recurrence, but not for the risk of death. Non-inferiority was more pronounced for taxane-only chemotherapy.

A recent, patient-level meta-analysis from EBCTCG, including around 18,000 patients from 15 trials, compared regimens including taxanes and anthracycline with taxane-only regimens. Patients receiving an anthracycline had a 14 % reduction in the risk of recurrence (risk ratio (RR) = 0.86, 95 % CI 0.79–0.93). Such benefit faded once only trials using anthracyclines sequentially to taxanes, rather than concurrently, were considered (RR 0.94, 95 % CI 0.83–1.06) [29]. Our results are not in conflict with the EBCTCG work. We also found that taxane-only chemotherapy was non-inferior to anthracycline-based chemotherapy in terms of recurrence risk, but we call for caution when interpreting our results as the between-study heterogeneity was high and we used study-level, aggregated data. The EBCTCG meta-analysis was not specifically designed for patients with TNBC. It only included the PATTERN study, while here we were able to identify two additional studies [23,27].

Carboplatin is being used for the neoadjuvant treatment of patients with TNBC, in combination with taxanes, with or without pembrolizumab [30,31]. For the same disease setting, several trials explored the impact of omitting anthracyclines in favour of a carboplatin and paclitaxel combination only. In the Neoadjuvant Study of Two Platinum Regimens in Triple Negative Breast Cancer (NeoSTOP) trial, patients receiving an anthracycline-free, taxane-based treatment regimen attained a similar residual cancer burden distribution, pathologic complete response rate and survival, compared to patients receiving standard, sequential chemotherapy [32]. Three of the eight studies included in the meta-analysis used a combination of carboplatin and a taxane as the adjuvant anthracycline-free arm. In all these studies, carboplatin plus paclitaxel proved to be superior or non-inferior compared to sequential chemotherapy. We did a meta-regression for the use of carboplatin as a moderator of the observed effects, excluding the studies using CMF and stratifying by type of experimental arm (i.e. only taxanes or taxanes plus carboplatin). We observed an improvement of the between-study heterogeneity (18 %) and although the regression coefficient was formally not significant, there was a trend towards larger effects (i.e. away from the NI margin) with the use of carboplatin. Therefore, we cannot rule out that risk estimates from studies using carboplatin may drive the observed treatment effect in favour of taxanes, but given the small number of studies, caution is needed (Supplementary Figure 1).

In our work, we could not systematically appraise the safety of the treatments under study due to the scarcity of data. Overall, reported patterns of adverse events were inconsistent (Supplementary Table 4). In some studies, the frequencies of grade three or four neutropenia [22,28,30], and peripheral neuropathy [19,21,22], were higher with anthracycline-free regimens compared to anthracycline-based regimens. A meta-analysis comparing sequential chemotherapy to six cycles of TC chemotherapy for patients with HER2-negative breast cancer found that using anthracyclines led to an increased risk of nausea, vomiting, mucositis, thrombocytopenia, and sensory neuropathy, but rates of febrile neutropenia, treatment discontinuation or drug-related deaths were comparable. Similarly, there was no difference in the incidence of heart failure, but the follow-up was relatively short, in the range of 3–6 years [33].

The studies varied widely in terms of study design, using both superiority or non-inferiority frameworks. Furthermore, only three of the eight studies included in the meta-analysis were specifically designed for patients with TNBC, while for the remaining five relevant risk estimates were available from subgroup analyses, which are inherently prone to bias. We addressed this obstacle by abstracting the unbiased raw event count for each study, treatment arm and outcome of interest. This allowed us to robustly estimate RRs and confidence intervals and to improve comparability.

We could only estimate risks for all stages combined as the distribution of events by tumour and nodal stage was not readily available. All the studies included in the meta-analysis allowed patients with nodal involvement or tumour larger than two cm. Based on current treatment standards, these patients would receive neo-adjuvant chemotherapy with or without immunotherapy, given the high risk of relapse. It is well-established that absolute risk reductions with adjuvant chemotherapy are larger for patients with high-risk disease than for patients with earlier stages of disease, while proportional risk reductions are similar and independent of tumour or nodal stage [4]. The inclusion of a high-risk patient population may skew the results away from the null in favour of anthracycline-based chemotherapy, as intensified treatment regimens by dose or schedule were found to bring additional benefit compared to standard regimens in terms of risk reduction [34]. Additionally, recent studies found that patients diagnosed with a stage IA disease had a breast-cancer-specific survival exceeding 90 % after five years from diagnosis. This population included patients with pT1a tumours who had favourable outcomes regardless of whether they received chemotherapy [35,36]. In women with small TNBCs, biomarkers such as stromal tumour-infiltrating lymphocytes (TILs) may help inform treatment decision-making. In a large retrospective series, patients with stage I TNBC, a TIL level higher than 50 %, and who forwent chemotherapy, experienced a disease relapse-free survival of 94 % or more [37]. Taken together, the impossibility of accounting for confounding by stage further restricts the clinical applicability of our findings.

Here we found that anthracycline-free regimens proved to be non-inferior for the risk of recurrence, regardless of the experimental arm. However, the effect estimate and the corresponding CI were statistically more robust (i.e. further from the NI margin) after only considering taxane-based experimental arms than when retaining all anthracycline-free regimens. In our systematic review, taxane-based chemotherapy was given for at least six months, equal to the duration of a sequential treatment. This is usually more than the four adjuvant TC cycles often proposed in clinical practice as an alternative, de-intensified treatment schedule [5,[38], [39], [40]]. Of note, here anthracycline-free chemotherapy proved to be potentially inferior to anthracycline-based chemotherapy concerning the risk of death. Our results suggest that anthracycline-free chemotherapy may be detrimental in the long term, while keeping in control the risk of relapse, in a patient population that typically experiences the highest risks in the first years after the diagnosis [4,29]. However, caution is needed as trials providing death counts were only six.

The definition of a non-inferiority margin is arbitrary and, as such, prone to bias. We decided to use published estimates from the 2012 EBCTCG meta-analysis on adjuvant chemotherapy, rather than expert opinion. Patient-level meta-analyses, in fact, amount to the highest level of quality of evidence and strength of recommendation [41]. We chose an NI margin that reflects the treatment effect of sequential chemotherapy compared to anthracycline-only chemotherapy, which was the standard adjuvant treatment regimen before the introduction of taxanes. We were only interested in gauging the potential detriment of omitting anthracyclines, as treatment effects in a superiority framework had already been made available by EBCTCG [29].

In conclusion, we found that anthracycline-free chemotherapy may be an option for patients with early TNBC. However, the evidence of non-inferiority was only for the risk of recurrence. Our findings warrant caution, as we were not able to account for the remarkable heterogeneity between studies, which included patient populations with varying stage distributions.

The relevance of our findings is limited to low-risk patients for whom upfront surgery followed by adjuvant chemotherapy is indicated, in light of the current recommendations on neoadjuvant chemotherapy. Our study suggests that sparing anthracyclines can be considered in selected patients, such as young women with small tumours or patients with comorbidities, or for patient preference. Overall, the expected incidence of adverse events is low, regardless of the chosen treatment regimen. Albeit rare, toxicities such as chronic heart failure or acute myeloid leukaemia may be life-threatening or have a prolonged impact on quality of life, as with taxane-induced peripheral neuropathy. These risks should also be weighed up when discussing the different adjuvant treatment options with the patients.

Further research is warranted, with randomised trials or prospective observational studies aimed at robustly assessing the impact of omitting anthracyclines in a homogeneous [6], low-risk population of patients with early TNBC. These studies should also incorporate a biomarker-driven stratification of the patient population using established prognostic factors such as TILs [42], to help identify patients to whom a treatment modulation strategy, including omitting chemotherapy, can be safely offered.

## CRediT authorship contribution statement

**Fabio Girardi:** Writing – original draft, Visualization, Validation, Software, Methodology, Formal analysis, Data curation, Conceptualization. **Caterina Barbieri:** Writing – review & editing, Validation, Methodology, Data curation. **Gaia Griguolo:** Writing – review & editing, Validation, Methodology. **Daniela Iannaccone:** Writing – review & editing, Data curation. **Christian Zurlo:** Writing – review & editing, Data curation. **Maria Vittoria Dieci:** Writing – review & editing, Validation, Conceptualization. **Valentina Guarneri:** Writing – review & editing, Supervision, Conceptualization.

## Funding

This research was funded by the Italian Ministry of Health Ricerca Corrente.

## Conflicts of interest

FG reports personal fees as invited speaker from AstraZeneca and Eli Lilly.

GG reports personal fees as invited speaker from Eli Lilly, MSD, and Novartis; advisory boards for Gilead, Menarini, and Seagen.

MVD reports personal fees for consultancy/advisory role from AstraZeneca, Daiichi Sankyo, Eli Lilly, Exact Sciences, Gilead, MSD, Novartis, Pfizer, Roche, Seagen.

VG reports personal fees for advisory board membership from AstraZeneca, Daiichi Sankyo, Eisai, Eli Lilly, Exact Sciences, Gilead, Merck Serono, MSD, Novartis, Olema Oncology, Pierre Fabre, Pfizer; personal fees as invited speaker for AstraZeneca, Daiichi Sankyo, Eli Lilly, Exact Sciences, Gilead, GSK, Novartis, Roche and Zentiva; personal fees for expert testimony from Eli Lilly. All reported COIs are outside the submitted work.
