# Supplementary material for: Omitting anthracyclines for the adjuvant treatment of patients with triple-negative breast cancer: A non-inferiority meta-analysis
Source: Breast. 2025 Jun 30;83:104524. doi: 10.1016/j.breast.2025.104524 (PMC12273482; doi:10.1016/j.breast.2025.104524)
Supplement: Multimedia component 1 [file mmc1.docx]

**Supplement**

**Supplementary Table 1**. Search strategy for Embase.

**Supplementary Table 2**. Search strategy for MEDLINE.

**Supplementary Table 3**. Search strategy for PubMed.

**Supplementary Table 4**. Risk-of-bias assessment according to the Cochrane’s Rob 2 tool.

**Supplementary Table 5**. Reported adverse events of interest from the studies included in the systematic review.

**Supplementary Figure 1**. Meta-regression for the effect of the use of carboplatin as a moderator.

| 1. | Exp Breast tumor/ or breast tumo*.mp. |
| --- | --- |
| 2. | Exp Breast Neoplasm/ or breast neoplas*.mp. |
| 3. | Breast cancer.mp. or exp breast cancer/ |
| 4. | Breast carcinoma.mp. or exp breast carcinoma/ |
| 5. | 1 or 2 or 3 or 4 |
| 6. | Adjuvant.mp. or exp adjuvant/ or exp cancer adjuvant therapy/ or exp adjuvant therapy/ or exp adjuvant chemotherapy/ |
| 7. | Postoperative.mp. or exp postoperative care |
| 8. | 6 or 7 |
| 9. | Exp anthracycline derivative/ or exp anthracycline/ or anthracycline*.mp. |
| 10. | Exp doxorubicin/ or doxorubicin.mp. |
| 11. | epirubicin.mp. or exp epirubicin/ |
| 12. | Exp taxane derivative/ or taxane.mp. |
| 13. | Paclitaxel.mp. or exp paclitaxel/ |
| 14. | Docetaxel.mp. or exp Docetaxel/ |
| 15. | 9 or 10 or 11 or 12 or 13 or 14 |
| 16. | Clinical trial.mp. or exp clinical trial/ or exp controlled study/ |
| 17. | Randomized controlled trial.mp. or exp randomized controlled Trial/ |
| 18. | Controlled clinical trial.mp or exp controlled clinical trial/ |
| 19. | 16 or 17 or 18 |
| 20. | Exp phase 2 clinical trial/ |
| 21. | Exp phase 3 clinical trial/ |
| 22. | 20 or 21 |
| 23. | 29 and 22 |
| 24. | 5 and 8 and 15 and 23 |

**Supplementary Table 1**. Search strategy for Embase; mp: multi-purpose; exp: exploded.

| 1. | Exp Breast Neoplasm/ or breast neoplas*.mp. |
| --- | --- |
| 2. | Breast tumo*.mp. |
| 3. | Breast cancer.mp. |
| 4. | Breast carcinoma.mp. |
| 5. | 1 or 2 or 3 or 4 |
| 6. | Adjuvant.mp. or exp Chemotherapy, Adjuvant/ or exp Chemoradiotherapy, Adjuvant/ |
| 7. | Exp Postoperative Care/ or postoperative.mp. |
| 8. | 6 or 7 |
| 9. | anthracycline.mp. or exp Anthracyclines/ |
| 10. | Exp Doxorubicin/ or doxorubicin.mp. |
| 11. | epirubicin.mp. or exp Epirubicin/ |
| 12. | taxane.mp. or exp Taxoids/ |
| 13. | Exp Paclitaxel/ or paclitaxel.mp. |
| 14. | Docetaxel.mp. or exp Docetaxel/ |
| 15. | 9 or 10 or 11 or 12 or 13 or 14 |
| 16. | Clinical trial.mp. or exp Clinical Trial/ |
| 17. | Controlled study.mp |
| 18. | Randomized controlled trial.mp. or exp Randomized Controlled Trial/ |
| 19. | Exp Controlled Clinical Trial/ or controlled clinical trial.mp. |
| 20. | 16 or 17 or 18 or 19 |
| 21. | Exp Clinical Trial, Phase II / |
| 22. | Exp Clinical Trial, Phase III/ |
| 23. | 21 or 22 |
| 24. | 20 and 23 |
| 25. | 5 and 8 and 15 and 24 |

**Supplementary Table 2**. Search strategy for MEDLINE; mp: multi-purpose; exp: exploded.

| 1. | Breast |
| --- | --- |
| 2. | Adjuvant |
| 3. | Postoperative |
| 4. | 2 or 3 |
| 5. | Anthracycline |
| 6. | Phase II |
| 7. | Phase III |
| 8. | 6 or 7 |
| 9. | Randomized |
| 10. | 1 and 4 and 8 and 9 |

**Supplementary Table 3**. Search strategy for PubMed.

| **Study** | **Randomisation process** | **Deviations from intended interventions** | **Missing outcome data** | **Measurement of the outcome** | **Selection of the reported results** | **Overall bias** |
| --- | --- | --- | --- | --- | --- | --- |
| **Earl HM** 2012 | **●** | **●** | **●** | **●** | **●** | **●** |
| **Rocca A** 2014 | **●** | **●** | **●** | **●** | **●** | **●** |
| **Mavroudis D** 2016 | **●** | **●** | **●** | **●** | **●** | **●** |
| **Najafi S** 2017 | **●** | **●** | **●** | **●** | **●** | **●** |
| **Wang J** 2019 | **●** | **●** | **●** | **●** | **●** | **●** |
| **Yu KD** 2020 | **●** | **●** | **●** | **●** | **●** | **●** |
| **Yu KD** 2021 | **●** | **●** | **●** | **●** | **●** | **●** |
| **Zheng F** 2021 | **●** | **●** | **●** | **●** | **●** | **●** |
| **De Gregorio A** 2022 | **●** | **●** | **●** | **●** | **●** | **●** |
| **Geyer CE**  2024 | **●** | **●** | **●** | **●** | **●** | **●** |

**Supplementary Table 4**. Risk-of-bias assessment according to the Cochrane’s Rob 2 tool. Colour code: green: low risk of bias; yellow: some concerns; red: high risk of bias.

|  |  | Acute leukaemia | |  | Cardiotoxicity (any grade) | |  | Neutropenia (grade 3 or 4) | |  | Febrile neutropenia | |  | Neuropathy (any grade) | |
| --- | --- | --- | --- | --- | --- | --- | --- | --- | --- | --- | --- | --- | --- | --- | --- |
|  |  |  |  |  |  |  |  |  |  |  |  |  |  |  |  |
|  |  | A-based | A-free |  | A-based | A-free |  | A-based | A-free |  | A-based | A-free |  | A-based | A-free |
|  |  |  |  |  |  |  |  |  |  |  |  |  |  |  |  |
| Earl HM |  | 3 | 0 |  | - | - |  | - | - |  | - | - |  | - | - |
| Rocca A |  | - | - |  | - | - |  | - | - |  | - | - |  | - | - |
| Mavroudis D |  | - | - |  | 1 | 1 |  | 11 | 54 |  | 2 | 2 |  | 7 | 17 |
| Najafi S |  | - | - |  | 2 | 1 |  | 1 | 3 |  | - | - |  | 1 | 2 |
| Wang J |  | - | - |  | - | - |  | - | - |  | - | - |  | - | - |
| Yu KD [PATTERN] |  | - | - |  | - | - |  | - | - |  | 30 | 3 |  | 3 | 12 |
| Yu KD [MASTER] |  | 1 | 0 |  | 13 | 3 |  | - | - |  | 10 | 11 |  | 3 | 15 |
| Zheng F |  | - | - |  | - | - |  | 67 | 100 |  | - | - |  | 45 | 25 |
| De Gregorio A |  | - | - |  | - | - |  | - | - |  | 114 | 145 |  | 45 | 23 |
| Geyer CE* |  | - | - |  | 0 | 0 |  | - | - |  | 37 | 74 |  | 46 | 18 |

**Supplementary Table 5**. Reported adverse events of interest from the studies included in the systematic review. Cells were left blank when the information for a specific adverse event was not available. *Data were available from an earlier publication; given that raw numbers were calculated from the reported percentages, inaccuracies may be possible.

**Supplementary Figure 1**. Meta-regression for the effect of the use of carboplatin as a moderator.
